# Regulation of Skeletal Muscle Plasticity by Protein Arginine Methyltransferases and Their Potential Roles in Neuromuscular Disorders

**DOI:** 10.3389/fphys.2017.00870

**Published:** 2017-11-01

**Authors:** Derek W. Stouth, Tiffany L. vanLieshout, Nicole Y. Shen, Vladimir Ljubicic

**Affiliations:** Department of Kinesiology, McMaster University, Hamilton, ON, Canada

**Keywords:** protein arginine methyltransferase, skeletal muscle, *in vivo*, cell culture, phenotype

## Abstract

Protein arginine methyltransferases (PRMTs) are a family of enzymes that catalyze the methylation of arginine residues on target proteins, thereby mediating a diverse set of intracellular functions that are indispensable for survival. Indeed, full-body knockouts of specific PRMTs are lethal and PRMT dysregulation has been implicated in the most prevalent chronic disorders, such as cancers and cardiovascular disease (CVD). PRMTs are now emerging as important mediators of skeletal muscle phenotype and plasticity. Since their first description in muscle in 2002, a number of studies employing wide varieties of experimental models support the hypothesis that PRMTs regulate multiple aspects of skeletal muscle biology, including development and regeneration, glucose metabolism, as well as oxidative metabolism. Furthermore, investigations in non-muscle cell types strongly suggest that proteins, such as peroxisome proliferator-activated receptor-γ coactivator-1α, E2F transcription factor 1, receptor interacting protein 140, and the tumor suppressor protein p53, are putative downstream targets of PRMTs that regulate muscle phenotype determination and remodeling. Recent studies demonstrating that PRMT function is dysregulated in Duchenne muscular dystrophy (DMD), spinal muscular atrophy (SMA), and amyotrophic lateral sclerosis (ALS) suggests that altering PRMT expression and/or activity may have therapeutic value for neuromuscular disorders (NMDs). This review summarizes our understanding of PRMT biology in skeletal muscle, and identifies uncharted areas that warrant further investigation in this rapidly expanding field of research.

## Introduction

Protein arginine methyltransferases (PRMTs) have emerged as powerful regulators of skeletal muscle plasticity. *In vitro* studies in muscle cells, as well as cell culture and *in vivo* investigations in muscle and non-muscle tissues have shown that PRMTs can stimulate or suppress molecules important for muscle remodeling by way of their specific methyltransferase activities. The majority of current research in skeletal muscle PRMT biology has focused on PRMT1, PRMT4 [also called co-activator-associated arginine methyltransferase 1 (CARM1)], PRMT5, and PRMT7, with only more recent studies being performed *in vivo* in rodent models. Indeed, PRMTs are involved in muscle development, regeneration, glucose metabolism, response to exercise, as well as neuromuscular disorders (NMDs). However, there is currently a gap in the literature regarding the direct roles of the various PRMTs during skeletal muscle remodeling. As such, further elucidation of PRMTs in skeletal muscle is required in order to advance our understanding of the impact that these molecules have in regulating phenotype determination, maintenance, and plasticity.

The purpose of this review is to provide a detailed survey of the state of knowledge regarding PRMT biology in skeletal muscle. We will first provide background on PRMTs, followed by a discussion on the role of PRMTs in regulating skeletal muscle phenotype. Furthermore, the potential clinical implications of PRMT biology in NMDs will be considered. Finally, we will conclude this with perspectives on PRMT functions in muscle, and close by proposing avenues for future research in the emerging area of PRMT-mediated skeletal muscle plasticity.

## Protein arginine methyltransferases

PRMTs are a family of enzymes that catalyze the addition of one or two methyl groups to the guanidine nitrogen atoms of arginine residues on target proteins, thereby altering the stability, localization, and/or activity of the marked molecules (Paik and Kim, 1968; Kakimoto, 1971). This post-translational modification of histones, transcription factors, and other proteins enables PRMTs to regulate many diverse cellular processes, such as gene transcription, mRNA splicing, DNA repair, signal transduction, protein subcellular localization, and cell cycle progression. PRMTs are generally ubiquitously expressed and the dysregulated expression or activity of these enzymes has been implicated in the progression of several prevalent health conditions, such as cancer and cardiovascular disease (CVD).

**Figure 1:**
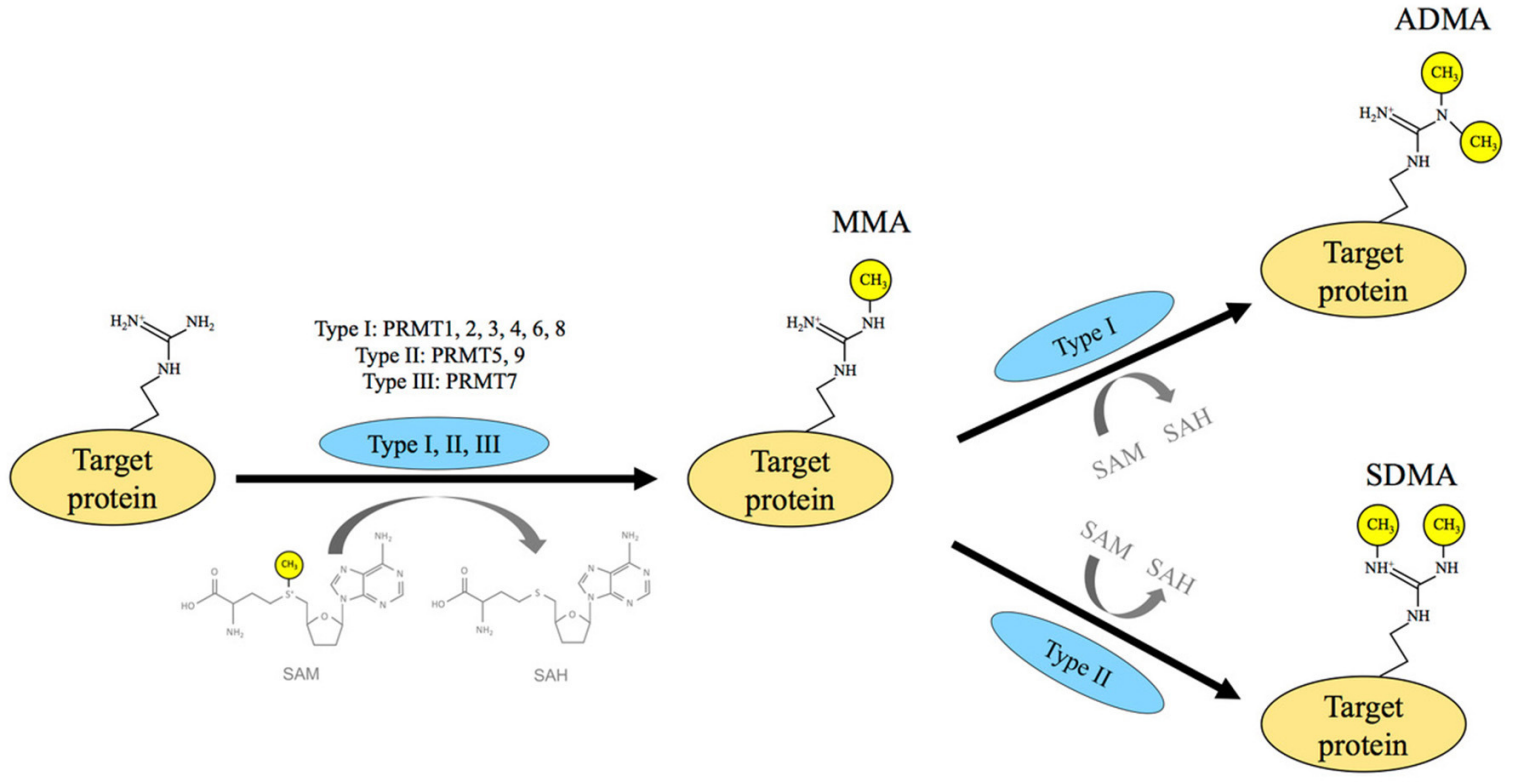
Protein arginine methyltransferase activity. Protein arginine methyltransferases (PRMTs) transfer a methyl group from S-adenosylmethionine (SAM) to the target protein and create the side product of S-adenosylhomocysteine (SAH). This enzyme family is classified into three categories based on their catalytic function: type I, II, and III. PRMTs in all three categories are responsible for the synthesis of the monomethylarginine (MMA) mark, with the type III enzyme PRMT7 having the sole responsibility of monomethylation. Both type I and II PRMTs monomethylate and dimethylate target proteins. Following monomethylation, type I PRMTs, which include PRMT1, −2, −3, −4, −6, and −8, deposit the asymmetric dimethylarginine (ADMA) mark, while type II PRMTs, including PRMT5 and−9, generate the symmetric dimethylarginine (SDMA) mark.

The PRMT family consists of nine members, all of which use S-adenosyl-L-methionine (SAM) as a methyl donor (Figure 1). SAM is generated by the enzyme methionine adenosyltransferase (MAT) using the substrates methionine and ATP (Gross et al., 1983). All PRMTs utilize SAM and L-arginine to catalyze the formation of the monomethylarginine (MMA) mark onto target molecules, which also results in the product S-adenosylhomocysteine (SAH). Proteins that contain glycine (G)- and arginine (R)-rich motifs and/or proline (P)-, glycine (G)-, and methionine (M)-rich regions are major targets for arginine methylation. In particular, arginines residing within GRG and PPPGMRPP sequences are preferred sites for PRMTs (Najbauer et al., 1993; Bedford et al., 1998; Cheng et al., 2007). Although PRMTs share many common features, they also have their own unique attributes. Among these, type I PRMTs, including PRMT1, −2, −3, −6, −8, and CARM1 catalyze the deposition of two methyl groups on one of the terminal nitrogen atoms of an arginine residue and produce the asymmetric dimethylarginine (ADMA) mark. In contrast, type II PRMTs, including PRMT5 and−9 generate the formation of the symmetric dimethylarginine (SDMA) mark by way of adding one methyl group to both terminal nitrogen atoms of an arginine residue. PRMT7 only catalyzes the formation of MMA and is classified as a type III PRMT. It is important to note that the cellular distribution of arginine methylation was analogous to other global modifications, such as phosphorylation and ubiquitination (Larsen et al., 2016).

PRMT1 and PRMT5 are the enzymes primarily responsible for generating ADMAs and SDMAs, respectively (Branscombe et al., 2001; Dhar et al., 2013). Notably, PRMT1 is the predominant PRMT in mammalian cells, and carries out >90% of total PRMT activity (Tang et al., 2000). Knockdown of PRMT1 expression results in significant compensatory increases in global MMA and SDMA levels mediated by other type I and II enzymes (Dhar et al., 2013). Whole body knockouts of PRMT2, −3, and −6 are viable and do not demonstrate an alternative phenotype (Swiercz et al., 2007; Iwasaki et al., 2010; Neault et al., 2012). In contrast, the complete loss of either PRMT1 or−5 are embryonic lethal, while CARM1 knockout mice die shortly after birth, indicating that these enzymes are critical for survival (Pawlak et al., 2000; Yadav et al., 2003; Yu et al., 2009; Kim et al., 2010; Tee et al., 2010; Table 1). Interestingly, the viability of full body PRMT7 null mice depends on the allele and genetic background (Ying et al., 2015; Blanc et al., 2016; Jeong et al., 2016).

**Table 1:** Skeletal muscle phenotypes in various murine PRMT knockout models.

**PRMT**	**Full-body knockout**	**References**	**Muscle stem cell-specific knockout**	**References**
PRMT1	Embryonic lethal	Pawlak et al., 2000; Yu et al., 2009	Impaired muscle regeneration subsequent to muscle injury	Blanc et al., 2017
CARM1	Born smaller and die shortly after birth	Yadav et al., 2003; Kim et al., 2010	Marked regeneration deficit following muscle injury	Kawabe et al., 2012
PRMT5	Embryonic lethal	Tee et al., 2010	Increased fibrosis and abolished muscle regeneration in response to muscle injury	Zhang et al., 2015
PRMT7	PRMT7 exon 3 knockout born with no overt phenotype but develop late-onset obesityPRMT7 exon 4 knockout born with short stature, predisposed to obesity and premature agingPRMT7 exon 5 knockout born with no overt phenotype but die shortly after birth	Ying et al., 2015; Blanc et al., 2016; Jeong et al., 2016	Defects in stem cell capacity to regenerate and self-renew after muscle injury	Blanc et al., 2016

*CARM1, co-activator-associated arginine methyltransferase 1; PRMT, protein arginine methyltransferase*.

PRMT gene expression is mediated, in part, at the transcriptional level. For example, studies *in vitro* and *in vivo* have shown that signal transducer and activator of transcription-6, nuclear factor kappa-light-chain-enhancer of activated B cells, and peroxisome proliferator-activated receptor gamma are among the factors that control PRMT1 transcriptional activation (Savoia et al., 2010; Liu et al., 2016). Furthermore, early growth response-1 and nuclear transcription factor Y have been identified as transcription factors that bind to promoters of CARM1 and PRMT5, respectively (Liu et al., 2014; Zhang et al., 2014). At the post-transcriptional level, PRMT1 pre-mRNA can be alternatively spliced to yield up to seven protein isoforms with varying catalytic activities and substrate specificities (Goulet et al., 2007). The existence of alternatively spliced isoforms for PRMT2, −7, and CARM1, has also been confirmed (Ohkura et al., 2005; Gros et al., 2006; Zhong et al., 2012). Further studies are required in order to gain a better understanding of the control of PRMT promoters, as well as how PRMT expression may be regulated post-transcriptionally, for example by spliceosomal processing, nuclear mRNA export, and/or mRNA stability.

PRMT function is regulated, in part, by its localization within the cell. In various non-skeletal muscle cell types, it is generally accepted that PRMT1 and PRMT5 are predominantly localized in the nucleus and cytosol, respectively (Herrmann and Fackelmayer, 2009; Tee et al., 2010). CARM1 is found primarily in nuclei where it serves as a transcriptional co-activator (Chen et al., 2002). Similar to CARM1, PRMT1 can also function as a transcriptional coactivator, whereas in contrast, PRMT5 has been identified as a general transcriptional repressor (Bedford and Clarke, 2009). Furthermore, to some extent, all PRMTs have an epigenetic function in nuclei. For instance, PRMT1 catalyzes the asymmetric arginine dimethylation of histone 4 arginine 3 (H4R3), whereas PRMT5 carries out symmetric arginine dimethylation of H4R3 and H3R8 (Wang et al., 2001; Pal et al., 2004). Asymmetric dimethylation via PRMT1 activates H4, whereas symmetric dimethylation by way of PRMT5 results in H4 repression (Feng et al., 2011). CARM1 modifies H3 by depositing the ADMA mark at R17 (Frietze et al., 2008). The specific histone marks catalyzed by PRMT1, −5, and CARM1, further distinguishes each enzyme from its PRMT family counterparts.

Regulation of PRMT activity can also be achieved through post-translational modification of the enzymes. Post-translational modifications of PRMTs include, but are not limited to, methylation, phosphorylation and glycosylation. Although automethylation activity has been reported for PRMT1 and CARM1, the functional impact of this specific reaction has yet to be fully elucidated (Gui et al., 2011; Kuhn et al., 2011). Recent work has demonstrated that phosphorylation by as yet unidentified kinases impair the methyltransferase activity of PRMT1 and CARM1 (Feng et al., 2009; Rust et al., 2014). On the other hand, protein phosphatase 2Ac binds directly to PRMT1 and inhibits its enzymatic activity, presumably via dephosphorylation (Duong et al., 2005). Furthermore, PRMT5 catalytic activity is disrupted when phosphorylated by the kinase JAK2 (Liu et al., 2011). Interestingly, O-linked N-acetylglucosamine transferase (OGT) modifies CARM1 by glycosylation (Cheung et al., 2008) and overexpression of OGT prevents phosphorylation of the enzyme (Sakabe and Hart, 2010). These results suggest that various post-translational modifications mediate PRMT methyltransferase activity. In any case, there is currently a gap in the literature regarding the identity of upstream molecules that modify PRMTs. In addition, a bona fide mammalian arginine demethylase remains to be confirmed (Yang and Bedford, 2013).

PRMT-binding proteins have the potential to regulate methyltransferase activity by activation, inhibition, or even through changing PRMT substrate specificity. For instance, B-Cell Translocation Gene 1 (BTG1) and BTG2 stimulate PRMT1 activity toward selected substrates (Lin et al., 1996). However, the mechanism by which this enzymatic activation occurs is not understood. In contrast, orphan nuclear receptor TR3 binds to the catalytic domain of PRMT1 and thereby inhibits PRMT1 methyltransferase activity (Lei et al., 2009). Both the nucleosomal methylation activator complex and the hSWI/SNF complex associate with CARM1 and PRMT5, respectively, thereby enhancing histone methylation (Pal et al., 2004; Xu et al., 2004). At the moment, the identity of other PRMT-binding proteins remains to be elucidated.

Altering PRMT expression and/or activity in conditions where PRMTs are dysregulated may have therapeutic value. Indeed, aberrant regulation of PRMTs is often associated with various diseases. For example, PRMTs are overexpressed in breast, prostate, lung, colon, and bladder cancers, as well as leukemia (Seligson et al., 2005; Cheung et al., 2007; Mathioudaki et al., 2008, 2011; Yoshimatsu et al., 2011; Gu et al., 2012; Zou et al., 2012; Baldwin et al., 2014). Knockdown of PRMTs via genetic technologies or pharmacological targeting inhibits proliferation of cancer cell lines *in vitro* (Yoshimatsu et al., 2011). Furthermore, PRMTs are mechanistically linked to the pathophysiology of endothelial dysfunction, atherosclerosis, uremia, and impaired immunological function via cellular events involving oxidative stress, autophagy, apoptosis, and inflammation (Tain and Hsu, 2017). The implications of PRMT biology in some of the most prevalent diseases of Western society are immense, which therefore underscores the importance of expanding our understanding of this family of enzymes. For more comprehensive surveys of the potential roles of PRMTs in cancer, CVD, neurodegenerative and metabolic diseases, interested readers are referred to a number of excellent reviews (Bedford and Clarke, 2009; Cha and Jho, 2012; Yang and Bedford, 2013; Wei et al., 2014; Morales et al., 2016; Blanc and Richard, 2017a).

## PRMTs in skeletal muscle

### *In vitro* studies

Our understanding of PRMTs in skeletal muscle expanded dramatically about 10–15 years ago thanks to a series of seminal studies that employed myogenic cell lines to investigate PRMT biology (Chen et al., 2002; Dacwag et al., 2007; Iwasaki and Yada, 2007). Examining myogenesis utilizing *in vitro* techniques is certainly an effective approach to study mechanisms of skeletal muscle remodeling. Indeed, even the more recent, comprehensive and elegant investigations of PRMTs in skeletal muscle make use of this versatile methodology (Kawabe et al., 2012; Zhang et al., 2015; Blanc et al., 2016, 2017). In this section, we survey the contributions that *in vitro* studies using myogenic cells have made to our progress in understanding PRMT biology in muscle and summarize the findings that have been particularly impactful in advancing knowledge regarding the roles PRMTs play in regulating skeletal muscle plasticity.

The first evidence alluding to a role of PRMTs in mediating skeletal muscle plasticity, specifically myogenesis, arose from the identification of CARM1 as a glucocorticoid receptor-interacting protein 1 (GRIP1) binding protein. (Chen et al., 2000). Here, GRIP1 and MEF2 were co-expressed in the nucleus during skeletal muscle differentiation. These initial findings led to an investigation that revealed that this methyltransferase was responsible for coactivating the transcription of myocyte enhancer factor-2C (MEF2C) via GRIP1 (Chen et al., 2002). Subsequent work demonstrated that CARM1 was required for later stages of myogenesis, as it is necessary for the binding of SWI/SNF Brg1 ATPase chromatin remodeling enzymes and myogenin to the myogenin promoter (Dacwag et al., 2009; Mallappa et al., 2011). Interestingly, decreased CARM1 protein content was reported throughout myogenesis (Kim et al., 2011), despite evidence of CARM1 having the greatest PRMT transcript levels in muscle cells (Wang et al., 2012). A comprehensive timecourse of CARM1 expression and activity during myogenesis is clearly necessary in order to assist in reconciling these disparate data. CARM1 may also play an important role in metabolic disease, as its expression and methyltransferase activity regulate a gene program involved in skeletal muscle glycogen metabolism (Wang et al., 2012).

The initial cell culture studies characterizing PRMT5 in muscle demonstrated that the enzyme was important for chromatin remodeling and the induction of myogenin (Dacwag et al., 2007; Paul et al., 2012). Interestingly, CARM1 and PRMT5 display both cooperative and differential functions during the muscle differentiation program. Similar to CARM1, PRMT5 also modifies the transcriptional activity of myogenic genes through association with the Brg1 ATPase subunit of SWI/SNF chromatin-remodeling enzymes (Dacwag et al., 2009). While PRMT5 is required for expression of the early gene MyoD, it is dispensable for subsequent expression of myogenin and MEF2D. After demonstrating that cooperator of PRMT5 (COPR5) binds to PRMT5 and histone 4, Paul et al. (2012) showed that C2C12 cells that lack COPR5 expressed very low levels of myosin heavy chain 1 and failed to form differentiated myotubes. As such, the PRMT5-associated protein COPR5 functions to coordinate the expression of cell cycle regulators in order for differentiation to proceed. Moreover, PRMT5 is required in muscle stem cells (MSCs) for both proliferation and differentiation (Zhang et al., 2015). While the mechanism(s) by which PRMT5 executes these functions remain elusive, recent evidence suggests that corepression of the cell cycle repressor p21 by PRMT5 is involved. Collectively, these *in vitro* studies confirmed that PRMT5 has well-established roles early in the myogenic process, most notable of which are its involvement in the proliferation of activated MSCs and the induction of myogenic determination.

The first report of PRMT1 biology in muscle revealed that the enzyme regulates the IR/IRS-1/PI3-K pathway involved in glucose transport in L6 skeletal muscle cells (Iwasaki and Yada, 2007). This enzyme is ubiquitously expressed within myocytes, being localized to the myonuclear, cytosolic, and sarcolemmal compartments (Iwasaki and Yada, 2007; Kim et al., 2011). More specifically, PRMT1 can be found in the cytoplasm and myonuclei of myoblasts before, during, and after fusion (Kim et al., 2011; Blanc et al., 2017), perhaps suggesting its multifunctional role during development in various sub-compartments of the cell. *Ex vivo* analysis of primary cells isolated from satellite cell-specific PRMT1 KO animals demonstrated enhanced proliferation, along with deficits in myogenic gene expression and cell morphology during differentiation (Blanc et al., 2017). These effects are mediated, in part, by the regulation of transcriptional activators Eya1/Six1 by PRMT1, which in turn control myoD expression (Blanc et al., 2017).

PRMT7 has been added to the list of PRMT family members involved in myogenesis. Evidence from *ex vivo* and *in vitro* cell culture studies have demonstrated premature senescence and delayed differentiation in PRMT7-deficient MSCs, coincident with a reduction in the size of the MSC pool (Blanc et al., 2016). Mechanistically, PRMT7 along with PRMT5, regulate the presence of Cdkn1a at the DNMT3b locus together with p21 expression, which is critical in preventing premature senescence (Blanc et al., 2016). Therefore, PRMT7 is ultimately required to preserve MSC regenerative and self-renewing capacity.

**Figure 2:**
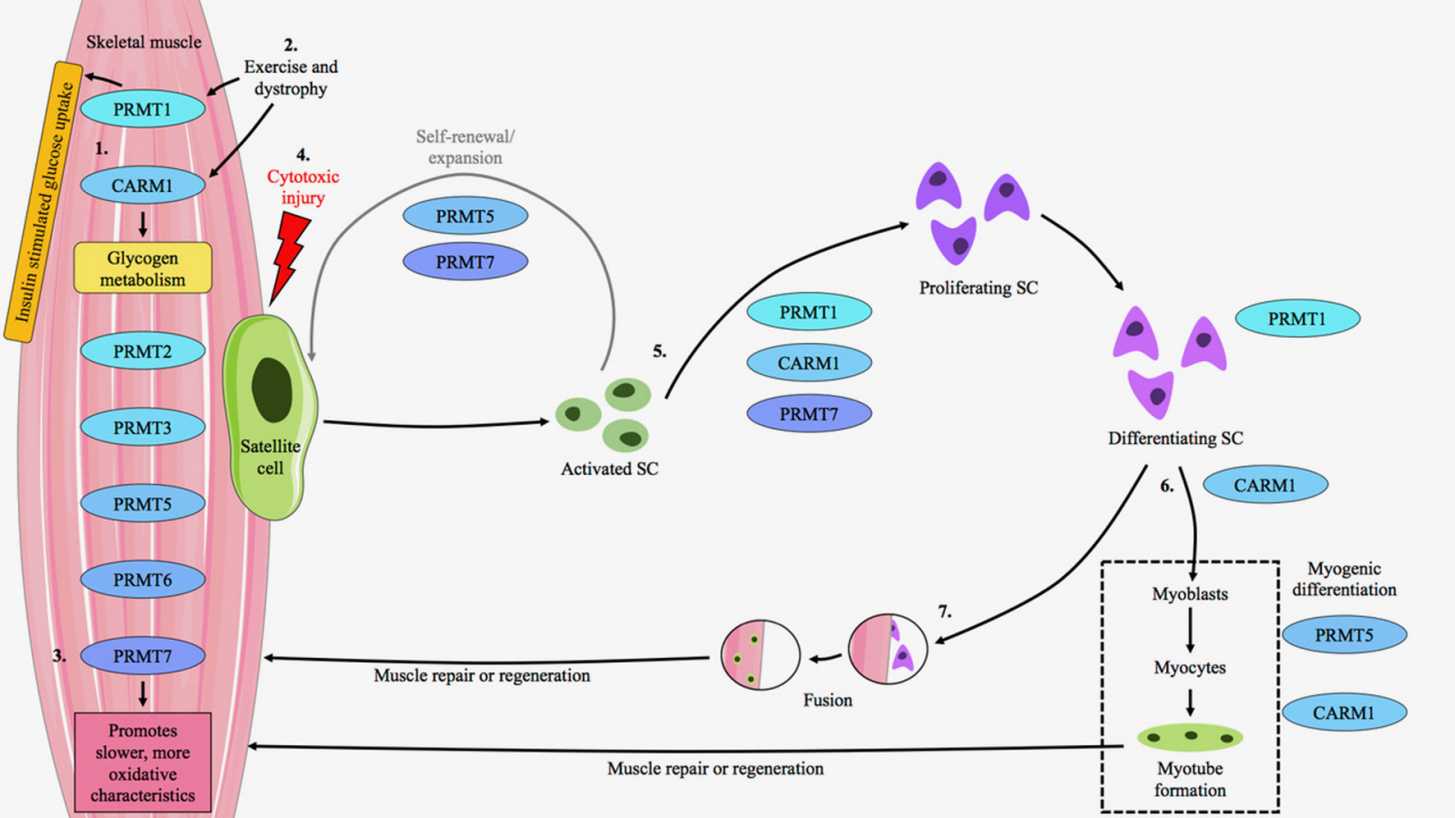
PRMT expression and function in skeletal muscle. PRMT1-7 are present within skeletal muscle at varying amounts depending, in part, on fiber type composition. (1) PRMT1 mediates insulin signaling and glucose disposal in skeletal muscle, while CARM1 is important for the regulation of glycogen metabolism. (2) PRMT1 and CARM1 transcripts are induced in response to exercise and muscular dystrophy. The protein content of these enzymes is also augmented in the muscle of mdx mice, a pre-clinical model of Duchenne muscular dystrophy. (3) PRMT7 regulates the slow, oxidative myogenic program, as its absence results in the expression of faster, more glycolytic characteristics. (4) The majority of PRMT research in skeletal muscle has examined their expression and function within the context of muscle repair and regeneration in response to cytotoxic injury via cardiotoxin (CTX) administration. In response to CTX, satellite cells (SCs) exit quiescence to enter the proliferative state and undergo expansion. PRMT5 and −7 are important during this process as they promote SC symmetric division and renewal. (5) CARM1, PRMT1, and −7 are critical for SC asymmetric division and further proliferation. PRMT1 is also important for terminal differentiation. (6) CARM1 facilitates myogenic differentiation, while PRMT5 is important for early myogenesis and CARM1 plays a role in the formation of mature myotubes. These cells aid in muscle repair or regeneration. (7) Alternatively, following differentiation SCs may fuse to existing myofibers to aid muscle repair or regeneration. Thus, PRMTs reside in skeletal muscle, their expression levels are modifiable, and they have important roles to play in muscle and SC biology.

In summary, *in vitro* and *ex vivo* cell culture studies have broadened our understanding of PRMT biology in regulating skeletal muscle plasticity, particularly during myogenesis (Figure 2). These important experiments have revealed that PRMT1, −5, and −7, along with CARM1 contribute to distinct, yet complementary, molecular milestones during the remodeling of skeletal muscle. A heightened understanding of the role of PRMTs in regulating skeletal muscle plasticity will continue to come from mechanistic studies employing cell culture techniques, as well as from *in vivo* works that emphasize the integrative biology of PRMTs in muscle.

### *In vivo* studies

Ljubicic and colleagues were the first to study PRMT biology in mammalian skeletal muscle *in vivo*. The authors observed that both the transcript and protein levels of PRMT1 and CARM1 were differentially expressed, and that acute and chronic conditions of muscle remodeling, namely exercise and dystrophy, respectively, altered gene expression of the enzymes (Ljubicic et al., 2012). This was followed by a study that examined the relative transcript levels of PRMTs 1–6 in mouse skeletal muscle (Wang et al., 2012). Analysis of PRMT mRNA content in muscles of varying fiber type composition, including the quadriceps (QUAD), soleus (SOL), and gastrocnemius (GAST) muscles, revealed fiber type-specific expression patterns of PRMT mRNAs. In the QUAD and SOL muscles, CARM1 was the most abundant transcript, followed by PRMT5 and PRMT1 (Wang et al., 2012). In contrast, CARM1 and PRMT5 were expressed at similar levels in the GAST, followed by PRMT1. Cumulatively, these initial *in vivo* studies demonstrated the presence of PRMTs in adult skeletal muscle and provided the first examples of PRMT gene expression during conditions of skeletal muscle plasticity.

Several studies have examined the roles of PRMT1, CARM1, PRMT5, and PRMT7 in regulating skeletal muscle regeneration and repair in response to cytotoxic injury (Kawabe et al., 2012; Zhang et al., 2015; Blanc et al., 2016, 2017). Recent, excellent surveys by Blanc and Richard summarize the contributions of these PRMTs to the *in vivo* myogenesis process (Blanc and Richard, 2017a,b). Notably, arginine methylation of Pax7 by CARM1 functions as a molecular switch controlling the induction of Myf5 during satellite cell asymmetric division and entry into the myogenic program (Kawabe et al., 2012). Work investigating the role of PRMT5 in MSC found that PRMT5 generates a ready state that keeps muscle satellite cells in standby, allowing rapid amplification when needed (Zhang et al., 2015). Furthermore, MSC fate is regulated, in part, through PRMT1-mediated arginine methylation within the Eya1/Six1/MyoD axis (Blanc et al., 2017). Finally, PRMT7 has been shown to be a regulator of the DNMT3b/p21 axis which is required to maintain MSC regenerative capacity (Blanc et al., 2016).

In addition to mediating muscle satellite cell biology, PRMT7 is a key regulator of the slow, oxidative myogenic program (Jeong et al., 2016). Muscles from whole body PRMT7 KO animals exhibit decreased oxidative metabolism concomitant with reduced expression of genes, such as peroxisome proliferator-activated receptor-γ coactivator-1α (PGC-1α), important for maintaining the slower, more oxidative phenotype (Jeong et al., 2016). These mice display an attenuated endurance exercise capacity compared to their wild type littermates, as well as decreased energy expenditure. In particular, PRMT7 regulates the slow, oxidative phenotype by interacting with the p38/ATF2/PGC-1α pathway, thereby enhancing PGC-1α expression and activity (Jeong et al., 2016). Collectively, while *in vivo* research elucidating the expression and function of PRMTs in muscle is still limited, recent work has clearly demonstrated the emerging importance of this family of enzymes as regulators of skeletal muscle plasticity (Figure 2).

## PRMT targets that could determine, maintain, and remodel skeletal muscle phenotype

PRMTs play a role in regulating tissue plasticity, in part, by altering the activity of several transcription factors and transcriptional coregulator targets. Various PRMT-interacting molecules, including PGC-1α, E2F transcription factor 1 (E2F1), receptor interacting protein 140 (RIP140), and tumor suppressor protein p53 (p53) are powerful regulators of skeletal muscle plasticity (Hawley et al., 2014). However, links between PRMTs and these phenotypic modifiers have not yet been explicitly made in skeletal muscle. The following section discusses these interactions in other cell types and thus provides some rationale for continuing their investigation in skeletal muscle.

### PGC-1α

PGC-1α is a transcriptional coactivator that interacts with multiple transcription factors to stimulate phenotype determination and remodeling programs in numerous tissues (Lira et al., 2010). In skeletal muscle, PGC-1α serves as a key regulator of the slow, oxidative myogenic program (Lira et al., 2010). For example, transgenic overexpression of the coactivator specifically within skeletal muscle results in mitochondrial biogenesis, a fast-to-slow myosin shift, structural and functional alterations in the neuromuscular junction, as well as improvements in VO2max and enhanced endurance capacity (Lin et al., 2002; Jäger et al., 2007; Calvo et al., 2008; Arnold and Salvatore, 2014). It is no wonder therefore, that exercise is a robust physiological stimulus for PGC-1α expression and activity in the skeletal muscle of rodents and humans (Mathai et al., 2008; Little et al., 2011).

A functional association between PRMT1 and PGC-1α was first discovered over a decade ago in CV-1 kidney cells (Teyssier et al., 2005). Notably, PRMT1-mediated methylation of the coactivator augmented PGC-1α transcriptional activity and mitochondrial biogenesis. Interestingly, PGC-1α methylation was found to be PRMT1-specific, since CARM1 did not enhance the coactivator function of PGC-1α. This seminal work clearly linked the most active PRMT with a master regulator of skeletal muscle phenotype determination, maintenance, and remodeling.

### E2F1

E2F1 regulates the expression of genes involved in cell proliferation and participates in the control of cell cycle progression (Blanchet et al., 2009). Whole body E2F1 knockout animals demonstrate a highly oxidative muscle phenotype, characterized by the increased expression of slower myosin isoforms, mitochondrial biogenesis, and enhanced fatigue resistance (Blanchet et al., 2011). In mouse embryonic fibroblast cells, PRMT2 represses E2F1 transcriptional activity in a manner dependent on its interaction with the retinoblastoma gene product (Yoshimoto et al., 2006). In addition, CARM1 is required for the estrogen-induced expression of E2F1 in a breast cancer cell line (Frietze et al., 2008). These studies indicate that E2F1 activity is in part regulated by PRMTs, providing potential linkage between PRMT-mediated arginine methylation and its potential impact on skeletal muscle plasticity.

### RIP140

RIP140 is a transcriptional corepressor for many nuclear receptors and transcription factors (Cavaillès et al., 1995; Horset et al., 1996; Wei et al., 2000, 2001). This molecule plays an important role in the regulation of skeletal muscle phenotype and metabolism by the suppressing the expression of phenotype modifying proteins, such as PPARβ/δ (Seth et al., 2007). RIP140 is expressed in a fiber type-specific manner, with low levels of the protein associated with a greater abundance of oxidative myofibers. Utilizing a variety of cell lines including COS-1, HEK293, 3T3-L1, and RIP140-null MEF, previous work demonstrated that arginine methylation suppresses RIP140 activity via two mechanisms. First, PRMT1-mediated methylation of RIP140 attenuates its interactions with histones (Mostaqul Huq et al., 2006). Second, methylation of the corepressor by PRMT1 promotes its nuclear export (Mostaqul Huq et al., 2006). Interestingly, whereas PRMT2 and −3, as well as CARM1 were all shown to interact with RIP140, PRMT2, and −3 could also modulate the repressive activity of RIP140, while CARM1 could not (Mostaqul Huq et al., 2006). These data indicate that PRMTs affect RIP140 localization and activity via selective interactions and/or modifications.

### p53

p53 plays a role in cell metabolism, growth and development (Vousden and Lane, 2007). Endurance-type exercise localizes p53 to the mitochondria in skeletal muscle where it induces Tfam transcriptional activity and stimulates organelle biogenesis (Saleem and Hood, 2013). p53 also promotes aerobic metabolism in skeletal muscle, plays a role in muscle differentiation, and may be a therapeutic target for diseases of mitochondrial etiology (Park et al., 2009; Yang et al., 2015; Safdar et al., 2016). Previous studies mechanistically revealed the involvement of PRMT1 and CARM1 in p53 activation (An et al., 2004). Utilizing *in vitro* techniques with H1299 lung carcinoma cells and U2OS osteosarcoma cells, prior evidence revealed independent and cooperative functions of p300, PRMT1, and CARM1 in mediating activation by p53 of its response element upstream of the GADD45 gene. More recent research demonstrated that PRMT5-mediated arginine methylation affects the target gene specificity of p53 (Jansson et al., 2008; Scoumanne et al., 2009). Additionally, PRMT5 depletion triggers p53-dependent apoptosis (Jansson et al., 2008).

**Figure 3:**
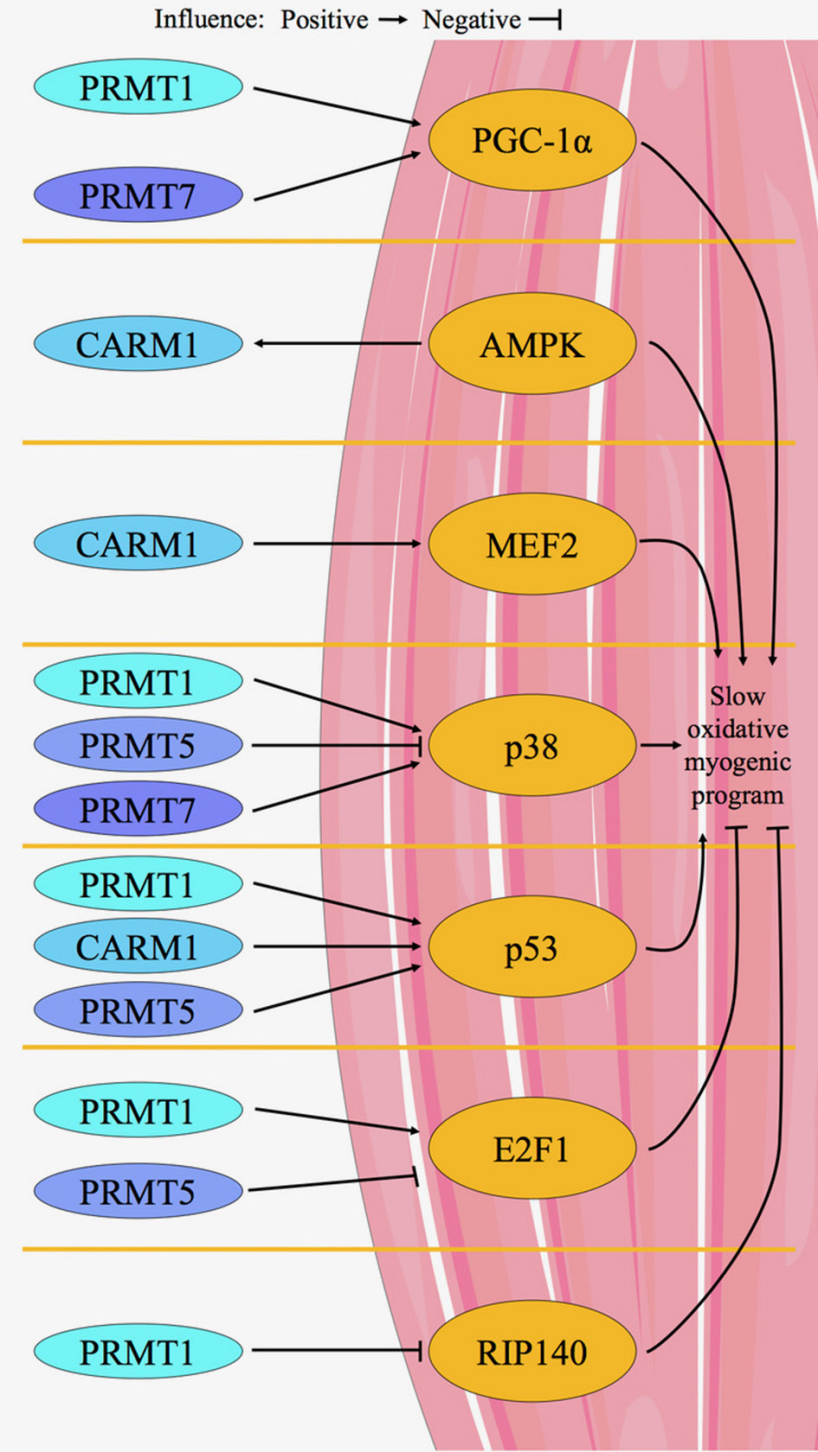
Proposed model for PRMT-mediated regulation of skeletal muscle plasticity. A limited number of *in vitro* and *in vivo* studies in skeletal muscle, as well as several other investigations in non-muscle tissues, provide the evidence for our model of PRMT-mediated control of muscle remodeling. CARM1, PRMT1,−5, and−7 target proteins that govern the determination, maintenance, and plasticity of skeletal muscle phenotype. For example, PGC-1α activity is enhanced by PRMT1 and−7. CARM1 is in a signaling axis with AMPK, and CARM1 directly influences MEF2 in muscle. p38 is stimulated by PRMT1 and−7, while PRMT5 inhibits its activity. p53 is activated by CARM1, PRMT1 and−5. PRMT5 inhibits E2F1, while PRMT1 activates the molecule. RIP140 is inhibited by PRMT1. Generally, chronic activation of PGC-1α, AMPK, MEF2, p38, and p53 cause a shift in skeletal muscle phenotype toward the slow, oxidative myogenic program, whereas in contrast E2F1 and RIP140 promote faster, more glycolytic characteristics.

In summary, numerous regulators of skeletal muscle phenotype maintenance and remodeling, including PGC-1α, E2F1, RIP140, and p53 are affected by PRMTs in non-muscle cells (Chang et al., 2010; Hua et al., 2013; Lim et al., 2013; Zheng et al., 2013; Kim et al., 2014; Park et al., 2014; Shin et al., 2016) (Figure 3). As the occurrence of protein arginine methylation is on par with that of phosphorylation or ubiquitylation (Yamagata et al., 2008; Garcia et al., 2011; Li et al., 2013; Larsen et al., 2016), it is reasonable to assume that there are many additional targets of PRMTs that have the potential to mediate muscle plasticity. Thus, continued investigation of the general role(s) of PRMTs in muscle, and more specifically their targets for interaction and methylation, is warranted.

## PRMTs and NMDs

PRMTs may be effective therapeutic targets for various NMDs, including Duchenne muscular dystrophy (DMD), spinal muscular atrophy (SMA), and amyotrophic lateral sclerosis (ALS). DMD is a progressive muscle wasting disease that causes the loss of muscle function due to the absence of dystrophin protein. PRMT1 and CARM1 expression levels are higher in dystrophic vs. healthy muscle (Ljubicic et al., 2012). In support of this, ADMA protein content is greater in dystrophic muscle (Mizobuchi et al., 1985). The elevated PRMT expression and activity in DMD may be an adaptive mechanism to counteract disease development. For example, this may be a compensatory upregulation to potentially mediate the phenotype-shifting abilities of PRMT targets PGC-1α and RIP 140 (Seth et al., 2007; Lira et al., 2010) toward the slow, oxidative myogenic program, which is more resistant to the dystrophic pathology (Ljubicic and Jasmin, 2013; Ljubicic et al., 2014). Moreover, during the cycles of degeneration and regeneration that characterizes DMD, PRMTs may enhance myogenesis and muscle repair (Kawabe et al., 2012; Zhang et al., 2015; Blanc et al., 2016, 2017). While these reports suggest a role for PRMTs in DMD, our understanding of PRMT biology in dystrophic skeletal muscle is still limited due to the dearth of studies in this area. Further work is required in order to determine whether PRMTs indeed attenuate the dystrophic pathology, and as such what the nature of PRMT-targeted therapies should be.

Similar to DMD, SMA is characterized by progressive muscle wasting (Sanchez et al., 2013). SMA is caused by the disruption of the survival motor neuron 1 (SMN1) gene, leading to reduced SMN protein levels and degeneration of spinal cord α-motoneurons (αMNs) and skeletal muscle (Markowitz et al., 2012). SMN has numerous critical functions in the cell that involve mRNA processing and transport (Coady and Lorson, 2011; Shukla and Parker, 2016). Many of these functions are mediated by SMN binding, via its Tudor domain, to protein targets that have been arginine methylated in a CARM1-dependent fashion (Cheng et al., 2007; Tadesse et al., 2008; Hubers et al., 2011). For example, the methylation of the splicing factor CA150 by CARM1 promotes the interaction between CA150 and the Tudor domain of SMN, which facilitates pre-mRNA splicing (Cheng et al., 2007). In SMA, SMN Tudor domain mutations that abolish interactions with methylated cellular proteins result in severe alterations in cell biology (Tadesse et al., 2008; Hubers et al., 2011). Furthermore, recent studies have demonstrated that CARM1 is abnormally upregulated in SMA, leading to the misregulation of a number of transcriptional, alternative splicing, and nonsense-mediated mRNA decay events, which very likely contributes to the SMA pathology (Sanchez et al., 2013, 2016). It comes as no surprise that PRMT5, another enzyme that interacts with SMN and is critical for homeostatic mRNA processing (Chari et al., 2009; Coady and Lorson, 2011), is also detrimentally affected in SMA (Boisvert et al., 2002). Indeed, examination of SMA patient cells revealed disrupted localization of proteins marked with SDMA, the product of PRMT5 activity (Boisvert et al., 2002). Altogether, these results clearly indicate that CARM1 and PRMT5 function are critical to SMA biology. Additional studies are necessary in order to establish the contribution of other PRMTs in SMA.

ALS is a progressive, life-limiting NMD that is characterized by the degeneration of αMNs and skeletal muscle (Yamaguchi and Kitajo, 2012). Familial and sporadic ALS can be provoked by mutations in the gene coding for fused in sarcoma/translocated in liposarcoma (FUS), an RNA-binding protein that regulates many steps in the RNA metabolism pathway (Taylor et al., 2016). In addition to this loss-of-function in RNA processing, FUS-ALS exhibits a toxic gain-of-function via the accrual of cytosolic inclusions of abnormal FUS proteins. PRMT1 interacts and methylates both wild type and mutant FUS proteins (Dormann et al., 2012; Tradewell et al., 2012; Yamaguchi and Kitajo, 2012; Scaramuzzino et al., 2013; Finelli et al., 2015; Tibshirani et al., 2015; Fujii et al., 2016). Tibshirani et al. (2015) observed that the redistribution of mutant FUS proteins to the cytoplasm led to the nuclear depletion of PRMT1, abrogating methylation of its nuclear substrates. They interpreted this loss of PRMT1 function as a consequence of the cytoplasmic accumulation of mutant FUS as contributory to the pathogenesis of FUS-ALS. Many other studies have also demonstrated that PRMT1-mediated arginine methylation regulates the nuclear-cytosolic shuttling of FUS (Yamaguchi and Kitajo, 2012; Scaramuzzino et al., 2013; Finelli et al., 2015; Tibshirani et al., 2015). In this role however, there are conflicting reports as to whether PRMT1 serves to exacerbate or alleviate FUS toxicity (Dormann et al., 2012; Tradewell et al., 2012; Yamaguchi and Kitajo, 2012; Scaramuzzino et al., 2013; Tibshirani et al., 2015; Fujii et al., 2016). For example, genetic ablation of the fly homolog of PRMT1 exacerbated the neurodegeneration induced by overexpression of FUS in a Drosophila model of FUS-ALS (Scaramuzzino et al., 2013), whereas PRMT1 knockdown in cultured murine motor neurons has differential effects on cytosolic FUS inclusion abundance, which depend, in part, on the timing and method employed to inhibit PRMT1 (Tradewell et al., 2012). Thus, PRMT1 plays a critical role in FUS-ALS by way of nuclear-cytosolic shuttling of FUS. Collectively, interventions that alter the expression and/or activity of PRMTs may offer an effective strategy for mitigating the severity and/or progression of DMD, SMA, and ALS. Further studies are required in order to elucidate the therapeutic potential of targeting these enzymes in NMDs.

## Conclusions and perspectives

Despite the limited number of studies investigating PRMT biology in skeletal muscle, recent evidence strongly suggest that this family of enzymes are important players in the regulation of skeletal muscle plasticity *in vivo*. PRMTs have been shown to mediate skeletal muscle development, regeneration, glucose metabolism, and oxidative metabolism. Experiments performed with non-muscle cell types have also revealed that powerful regulators of muscle phenotype determination, maintenance, and remodeling, such as PGC-1α, E2F1, RIP140, and p53 are downstream targets of PRMTs. It would be logical to determine whether these putative PRMT targets are in fact PRMT-interacting molecules in skeletal muscle, and if so, what are the functional consequences of these interactions. Although full-body murine knockout experiments have firmly established the importance of PRMTs for survival, the functional role(s) of PRMTs during skeletal muscle remodeling remains unclear. As such, investigations utilizing skeletal muscle-specific PRMT knockout or overexpressing animals in experiments that elicit muscle remodeling will assist in addressing this knowledge gap. Complementary strategies, such as adeno-associated virus-mediated overexpression or RNA knockdown of select PRMT enzymes in discrete skeletal muscles of defined function, would also assist in the elucidation of the role(s) of PRMTs in this tissue. Importantly, the expression and/or functions of PRMTs are dysregulated in various NMDs. The notion that PRMTs are logical pharmacologic and/or physiological targets for NMDs, such as DMD, SMA, and ALS, raises the question of whether PRMTs attenuate or enhance the pathology. Proof-of-principle studies that cross skeletal muscle-specific PRMT transgenic mice with pre-clinical murine models of these NMDs will be advantageous in resolving this uncertainty. Moreover, future research that aims to identify molecules that mediate (1) PRMT gene expression by, for example regulating PRMT promoter activity, as well as (2) methyltransferase functions, for instance via post-translational modification of PRMTs, is warranted. In conclusion, PRMTs have clearly emerged as critical regulators of skeletal muscle plasticity. The continued examination of this family of enzymes will expand our understanding of the molecular mechanisms that govern muscle phenotype determination, maintenance, and remodeling. This will also inform novel, PRMT-based therapeutic approaches for the most prevalent NMDs.

## Author contributions

VL designed the outline of the paper; DWS, TLvL, NYS, and VL wrote the paper.

### Conflict of interest statement

The authors declare that the research was conducted in the absence of any commercial or financial relationships that could be construed as a potential conflict of interest.
